# Strategies for Engineering Virus Resistance in Potato

**DOI:** 10.3390/plants12091736

**Published:** 2023-04-22

**Authors:** Jiecai Liu, Jianying Yue, Haijuan Wang, Lingtai Xie, Yuanzheng Zhao, Mingmin Zhao, Hongyou Zhou

**Affiliations:** 1 College of Horticulture and Plant Protection, Inner Mongolia Agricultural University, Hohhot 010018, China; 2 Inner Mongolia Academy of Agricultural and Animal Husbandry Sciences, Hohhot 010031, China

**Keywords:** potato, viral resistance, engineering, RNAi

## Abstract

Potato (*Solanum tuberosum L.*) is an important vegetable crop that plays a pivotal role in the world, especially given its potential to feed the world population and to act as the major staple food in many developing countries. Every year, significant crop loss is caused by viral diseases due to a lack of effective agrochemical treatments, since only transmission by insect vectors can be combated with the use of insecticides, and this has been an important factor hindering potato production. With the rapid development of molecular biology and plant genetic engineering technology, transgenic approaches and non-transgenic techniques (RNA interference and CRISPR-cas9) have been effectively employed to improve potato protection against devastating viruses. Moreover, the availability of viral sequences, potato genome sequences, and host immune mechanisms has remarkably facilitated potato genetic engineering. In this study, we summarize the progress of antiviral strategies applied in potato through engineering either virus-derived or plant-derived genes. These recent molecular insights into engineering approaches provide the necessary framework to develop viral resistance in potato in order to provide durable and broad-spectrum protection against important viral diseases of solanaceous crops.

## 1. Introduction

Potato (*Solanum tuberosum L.*) is an important solanaceous food crop. It has the potential to feed the populating world and especially to act as the major staple food in many developing countries. Compared with other food crops, potato contains more nutrition reagents, including proteins, carbohydrates, and minerals [1]. The human need for food safety drives the high-quality development of potato and has provided many ways to meet rising food demands, especially in food-deficit countries.

However, an important problem in potato production is the degradation of seed potatoes caused by viral diseases, which has been an important factor restricting potato production for a long time. After infection by viral diseases, symptoms on leaves or tubers such as necrotic mosaic and overall stunted growth appear, which can result in yield decreases and poor-quality tubers. Commonly, potato production losses caused by viral infection in potato can reach up to 20~30% with serious production reductions of more than 80%.

Up to now, around 40 different viruses and 2 viroid species have been known to infect potato [2]. Among them, potato virus Y (PVY; genus *Potyvirus*), potato leafroll virus (PLRV; genus *Polerovirus*), and potato virus X (PVX; genus *Potexvirus*) are the most important viruses that cause significant potato production losses worldwide [3,4,5]. Once these viruses invade potato plants or tubers, they exhibit a variety of degradation types, including mosaic, such as leaf curl and necrosis, bundle top, plant dwarfing, and leaf yellowing. Young leaves show discoloration and shrinkage. Tubers become small, cracked, and pointed; show internal network necrosis; and in most cases lose germination ability and cannot be planted. There is a significant difference between these viruses and other pathogens; that is, after the virus particles enter the plant body with the help of other factors (such as insects, plant wounds caused by humans, natural factors, etc.), they use the information, energy, and enzyme systems of plant cells to complete the replication and proliferation of the virus itself. This plant virus proliferation mechanism brings great difficulty to the prevention and control of viral diseases.

At present, although virus-free seed potato used in production can reduce the damage of virus disease, virus reinfection in the field in the middle and late growth stages can also lead to a significant reduction in yield. Moreover, although virus-free seed potato technology has become well established, some viruses (such as PVS) are difficult to remove, leading to the need to manually remove infected plants in the production of field seed potato, which is time-consuming and costly.

Potato is a hetero-tetraploid plant, and it is very difficult to develop antiviral varieties via conventional breeding methods. Cultivating virus-free seed potato through stem tip detoxification is an effective preventive measure in controlling potato viruses. However, plants may still be infected by various viruses during the growth seasons, sometimes even to the extent of epidemic disease.

With the rapid development of molecular biology and plant genetic engineering technology, generating crops with enhanced viral resistance has become a reality. Virus resistance in potato has been engineered through different approaches via traditional plant breeding and genetic engineering [1]. Virus resistance in plants has been obtained through the transgenic expression of viral proteins and non-viral factors. These strategies will lead to highly effective and broad-spectrum resistance to virus disease in plants [6]. Notably, RNA interference (RNAi)-mediated resistance targeting the viral coat protein (CP) of PVY, PVX, PLRV, and potato virus S (PVS) in potato has been reported [7] to confer resistance [8,9]. At the application level, genetically modified (GM) potatoes, including virus-resistant potatoes generated through genetic engineering, are currently being incorporated and commercialized in some countries [10].

## 2. Engineering Virus-Derived Viral Resistance in Potato

Since virus-resistant transgenic tobacco was obtained by transforming the CP gene of the tobacco mosaic virus (TMV) [11], the antiviral genetic engineering of plants has developed rapidly. With respect to potato antiviral gene engineering, researchers have made some progress in exploring the viral CP gene, viral protein gene, viral replicase gene, and viral RNA to create genetically engineered antiviral potato germplasm.

Given that CP-mediated resistance to viruses has represented one of the successes of plant genetic engineering [6], the CP gene of some potato viruses, such as PVY, PVX, and PLRV, has been cloned and transferred into potato successively to obtain virus-resistant potato plants [12,13,14]. In certain cases, resistant plants have been applied in the field for several years.

Viral CP has a variety of functions, including the ability to wrap the nucleic acid of a virus and to determine the host range of infection. The application of viral CP genes in potato antiviral gene engineering is based on viral CP genes inhibiting virus uncoating so as to block virus infection. Recently, it has been found that CP can bind to the nucleus and acts as a trans-acting factor to regulate the expression of nuclear genes so that the virus can successfully complete assembly in the host cytoplasm. In addition, CP-mediated viral resistance is caused by an important mechanism of cross-protection in potato.

In most cases, this antiviral ability is only against the CP-donor virus or a few strains of the virus that are very closely related. Moreover, CP-mediated resistance often only delays infection time and cannot achieve complete antiviral ability to resist. Plants transformed with the CP gene are only protected from low doses of the virus. Once the viral vaccination is changed, plants become not at all resistant to viral infection. This places a strict limit on field application.

The application of viral CP genes could also become a problem with respect to virus transmission where plants are transformed with the CP of a virus spreading via an insect vector in the field. It is reported that transgenic CP can encapsidate heterologous viral RNAs by which it may help the virus to gain aphid transmission ability [15,16]. For example, the transgenic expression of the CP of PLRV can encapsulate and promote the aphid transmission of viroid RNAs. In some cases, the transgene or its RNA product can recombine with an inoculated virus to generate a variant with novel biological properties [17]. To overcome these problems, efforts have been made to improve coat-protein-mediated virus resistance by combining different viral CP in the same plant or by conjugating coat protein genes with satellite RNA into plant cells to obtain a broader antiviral spectrum.

Replicase is an RNA polymerase encoded by viral genes that can specifically synthesize the positive- and negative-strand RNA of viruses. Functionally, it is similar to RNA-dependent RNA polymerase (RdRp). During viral replication, replicase utilizes the plus strands of viral RNA as a template to synthesize negative-stranded RNA and then uses negative-stranded RNA as a template for synthesizing plus-stranded RNA. In most viruses, replicase is a replicase complex formed by the combination of virus-encoded proteins and host components. In one study, highly PVX-resistant transgenic potatoes were obtained by introducing partial or full-length replicase genes of PVX [18]. Further, transgenic potatoes with the full-length sequence of the PVY NIb gene with 381 deletions at the 5’ end and the antisense RNA of NIb were generated and showed high PVY resistance [19]. To obtain PLRV-resistant plants, the 3′ terminal sequence and 5′ terminal sequence of the potato leaf roll virus (PLRY) replicase gene (ORF2b) were introduced into potato.

Studies have shown that the transcription of a gene with the deletion of replicase can mediate viral resistance, but resistance is far stronger when the deletion of the RNA transcription is translated into the deletion of the replicase.

Compared with resistance mediated by CP, resistance mediated by replicase has been found to be stronger, showing resistance to high concentrations of virion and viral RNA (500 μg/mL). However, replicase-mediated resistance is more specific; that is, the replicase gene of a virus transferred into plants is only resistant to the same virus but not to another strain. Since plant RNA viruses mutate quickly and easily produce different strains, it is difficult to use replicase-mediated resistance in the field.

Antisense RNAs (asRNAs) are complementary to messenger RNA (mRNA) strands transcribed within cells [20]. asRNAs occur in nature but normally cannot be detected. However, synthetic RNAs directed at specific targets have been widely studied for their inhibition of gene action. The effect of antisense RNA occurs mainly in transcription as well as in the processing of transcripts. Antisense RNA for acquiring antiviral infection capability and protecting plants from systemic infection has been successfully established. Antisense RNA techniques that aim to encode templates can be applied to many viruses, especially those whose product is spread by aphids or whose infection is limited to specific tissues, such as PLRV. Transgenic potato plants expressing complementary RNA with the PLRV CP gene have shown similar resistance to viral infection as transgenic plants expressing the PLRV CP gene. Lindbo and Dougherty believe that transcription accumulation leads to further replication in the middle of righteous RNA interference in negative replication [21].

Since antisense transcriptions cannot be transferred to the cytoplasmic replication region, the antisense RNA of CP is difficult to use against highly effective viruses such as PVY. Due to the insufficient expression of antisense RNA, antisense RNA-directed resistance is weak, which leads to the unsatisfactory application of antisense RNA technology to obtain antiviral infection in practice. Nevertheless, it is worth remaining open to the possibility of improving the expression amount of antisense RNA.

## 3. Engineering Virus-Resistant Plants Using Plant Endogenous Genes in Potato

With the exploration of host–virus interaction, more scientists have become focused on the engineering of virus-resistant plants using plant endogenous genes. At present, antiviral genes of potato have been found in both wild and cultivated species, which are usually divided into extreme resistance (ER) genes and hypersensitive resistance (HR) genes. ER genes are resistant to many viruses and can effectively prevent the reproduction of viruses in the early stages of infection. Evidence has shown that plants expressing ER typically will remain symptomless and experience extremely low viral accumulation in inoculated leaves [22,23], whereas HR can be activated to effectively restrict pathogen growth during host as well as non-host interactions [24]. The HR gene is resistant to various virus species and is a quick defense response to local cell necrosis after virus infection, limiting the further expansion of the virus. Host resistance to both ER and HR in potato has been recognized against PVY [25,26,27]. In potato cultivars, Ry genes confer ER to all PVY strains. The *Rysto* gene (located on chromosome XII) from *S. stoloniferum* [28,29], the *Ryadg* gene (located on chromosome XI) from *S. tuberosum* ssp. andigena [30], and the *Rychc* gene (located on chromosome IX) from *S. chacoense* [31] were identified to confer ER, and the *Rychc* gene was also found to confer extreme resistance to PVY [32]. In addition, the genes *Ryadg*, *Rysto,* and *Rychc* derived from other potato cultivars such as *S. tuberosum* subsp. *andigena* Hawkes, *S. stoloniferum* Schlechtd. Et Bché., and *S. chacoense* Bitt., respectively, have been used in potato breeding programs [25,31,33,34,35,36].

In exploring how PVY CP is recognized by Rysto, it has been demonstrated that Rysto associates directly with central 149 amino acids of the CP domain in PVY [37]. Each deletion mutant of the CP core region affects the ability of Rysto to trigger defense. The appropriate folding of the CP core is crucial to Rysto-mediated recognition [37]. This sheds light on its potential utility in engineering virus resistance in various crops. The *Y-1* gene was identified in *S. tuberosum* ssp. *andigena* and was found to be recognized by PVY, inducing cell death without preventing the systemic spread of PVY in potato [38]. Moreover, the LRR or other regions of the *Y-1* gene might be developed into a useful resistance gene for potato breeding. *Y-1* is located in potato chromosome XI in an *R* gene cluster, which includes the gene *Na* for HR to PVA and the gene *Ryadg* for ER to PVY [23,30,39]. More recently, gene *G-Ry,* a homolog of *Y-1,* was isolated and observed to enhance resistance to PVY [40].

In potato, strain-specific *Ny* genes in several popular potato cultivars have exhibited HR against PVY [41,42,43,44]. *Ny-1* from potato cultivar Rywal, a hypersensitivity gene, confers HR against both common and necrotic strains of PVY. Similar to various resistance genes in *solanaceous* genomes, *Ny-1* was mapped on the short arm of potato chromosome IX. The expression of HR was temperature-dependent in potato cultivar Rywal. Strains PVY_O_ and PVY_N_ and subgroups PVY_NW_ and PVY_NTN_ were effectively restricted in plants at 20 °C. When plants were grown at 28 °C, viruses could systemically spread but without symptoms [41,43]. In field trials, PVY was restricted to inoculated leaves, and PVY-free tubers were produced [41,43]. Further, an HR gene *Ny-2* conferring resistance to PVY was mapped on potato chromosome XI in potato cultivar Romula [44].

The *Nytbr* gene was identified on chromosome IV, although this location was not consistent with any other resistance genes in potato [45]. Nytbr was identified in a cross between *Solanum tuberosum* and *Solanum berthaultii* segregated for monogenic-dominant hypersensitivity to PVY. Plants bearing *Nytbr* displayed necrosis symptoms upon PVY infection. Benoît Moury et al. demonstrated that the helper component proteinase (HC-Pro) cistron of PVY induces hypersensitivity and resistance in potato genotypes carrying dominant resistance genes on chromosome IV [46]. They found that the *Nc(tbr)* and *Ny(tbr)* genes in *Solanum tuberosum* determine HR against clade C and clade O of PVY, respectively, via necrotic reactions and the restriction of virus systemic movement, whereas a dominant gene, *Nc(spl)*, was mapped on potato chromosome IV close or allelic to *Ny(tbr)* and conferred a resistance to *S. sparsipilum* with the same phenotype as *Nc(tbr)*. The HC-Pro cistron of PVY was shown to affect necrotic reactions and resistance in plants carrying *Nc(spl)*, *Nc(tbr)*, and *Ny(tbr)*. However, inductions of necrosis and of resistance to systemic virus movement in plants carrying *Nc(spl)* were determined by different regions of the HC-Pro cistron [47]. Moreover, genomic determinants outside the HC-Pro cistron are involved in the systemic movement of PVY after the induction of necroses on inoculated leaves of plants carrying *Ny(tbr)*. It seems that *Ny(tbr)* resistance may have been involved in the emergence of PVY isolates through a recombination breakpoint near the junction of HC-Pro and P3 cistrons in potato crops. Thus, this might serve to explain virus resistance breakdown caused by recombination other than the accumulation of nucleotide substitutions [43].

Further, it was demonstrated that the gene Ny in potato is responsible for PVY overcoming or triggering hypersensitive resistance to PVY strain group O [48]. For example, the residues 227 to 327 of HC-Pro are the viral determinants for overcoming Nytbr resistance. This HC-Pro region with eight residues and a special three-dimensional conformation model in PVY^N^ differs from PVY^O^ strains, suggesting a structure–function relationship in recognition of PVY^O^ HC-Pro by *Nytbr*.

In response to infection by PVX, the *Rx1* gene mediates ER, and viral replication is rapidly terminated, which results in symptoms such as cell death and lesion formation in plants [49]. *Rx1*′s ER is conserved in *Nicotiana benthamiana (N. benthamiana)* by the evidence of a strong hypersensitive response in *Rx1*-overexpressed plants [49]. Moreover, Townsend et al. identified a golden-like transcription factor that interacts with *Rx1* and mediates antiviral immunity, which enables the nonspecific DNA-binding *Rx1* to confer ER to PVX [50].

PLRV is one of the most important virus diseases in potato and is widespread across the world [51]. A quantitative trait locus (QTL) analysis of resistance to PLRV virus accumulation revealed one major and two minor QTLs [52]. The major QTL (*PLRV.1)* was mapped to potato chromosome XI in a resistance hotspot containing several genes for qualitative and quantitative resistance to viruses and other potato pathogens with 50% and 60% phenotypic variance. The two minor QTLs were mapped to chromosomes V and VI. Those genes with sequence similarity to the tobacco *N* gene for resistance to TMV were found to be tightly linked to *PLRV.1*. Based on the cDNA sequence of an *N*-like gene, the sequence-characterized amplified region (SCAR) marker Nl271164 was developed to select potatoes with resistance to PLRV [52].

These identified genes associated with potato viral resistance (Table 1) can be used for antiviral breeding and to create potato varieties with resistance to a virus or a variety of viruses. However, scientists should make further efforts to bring about either resistance gene application or the discovery of new resistance genes in potato.

## 4. RNAi-Mediated Viral Resistance in Potato

RNA silencing is a common gene-regulation mechanism in eukaryotes, functionally involving various biological processes, including the defense against viruses [58,59]. Small interfering RNAs (siRNAs) of 21–24 nt in length, initially processed by Dicer-like (DCL) endonucleases, are the core effectors in this immune system [60,61]. Basically, one strand of the sRNA duplex is recognized by one of the AGO family proteins, forming an RNA-induced silencing complex (RISC) [62]. DCL4 and DCL2 generate 21 and 22 siRNAs, respectively, from the intermediates of double-strand RNAs (dsRNAs) during viral replication, which mediate defenses against RNA viruses through siRNA-directed and AGO-mediated cleavage and the degradation of viral RNA [63]. By contrast, RNA-dependent RNA polymerase (*RDRs)* can convert aberrant single-stranded RNA into dsRNA precursors of secondary siRNAs to reinforce RNAi [58,64]. As an effect on the immune system, RNAi offers a very promising approach for genetically engineering resistance against viruses in transgenic plants. The first layer of the antiviral system of RNA silencing is the DCL-mediated cleavage of the initial trigger viruses. DCL4 plays a major role in antiviral silencing against plus-strand RNA viruses, while DCL2 has a subordinate role when DCL4 is inhibited. DCL3 makes a minor contribution to the antiviral process [65].

It has been demonstrated that RNA silencing plays an important role in viroid infection in plants. The stable structure of viroids serves as the dsRNA substrate for host Dicer-like enzyme cleavage to produce biologically active small RNAs that gain resistance to RISC-mediated degradation [66]. For example, the replication of the potato spindle tuber viroid (PSTVd) in infected tomato plants was found to induce resistance to RNA silencing, although viroid-specific siRNAs were biologically active in guiding the RISC-mediated cleavage. This suggests that the PSTVd secondary structure might play a crucial role in resistance to RNAi [66]. Another possibility is that some viroids may build up a structure to avoid DCL cleavage in order to infect plants; this structure may change to become more accessible to RISC complexes and AGO targeting.

It has been reported that RNAi plays an unexpected beneficial role in viroid titer. DCL4 may have a positive effect on PSTVd accumulation in *N. benthamiana*, while DCL2 does not. However, the reason for this effect remains unknown. It appears that the generation of sRNAs from viroids is complicated and possibly involves multiple DCL pathways. RDR6-dependent RNA silencing pathways are linked to viroid-induced pathogenesis. Ta-siRNA biogenesis and the replication processes of members of the family Pospiviroidae share several similarities. This indicates that disease symptoms might result from the incorporation of viroid replication intermediates into the ta-siRNA biogenesis pathway. The interaction of viroids and RNAi might be useful in designing the targets of engineering viral resistance.

siRNAs are usually produced from long dsRNAs and miRNAs originated through the nucleolytic maturation of miRNA genes (MIR) with a self-complementary fold-back structure [67]. Precise excision from the stem of the fold-back precursor yields a duplex intermediate (miRNA/miRNA*) that ultimately promotes the miRNA strands to RISC [68]. Vaucheret et al. demonstrated that exchanging the miRNA/miRNA* sequence within a premiRNA does not affect its biogenesis as long as the secondary structure of the precursor is kept intact [69], which makes it possible to modify miRNA sequences to create artificial miRNAs (amiRNAs) that can target specific sequences. Plant miRNA precursors have therefore been engineered to target one or several interested genes to provide highly specific and effective post-transcriptional gene silencing (PTGS) in plants [70]. Moreover, Simón-Mateo proposed that viruses could be targets of miRNA-mediated silencing [71], which has opened up the possibility of engineering amiRNAs against viral infections. In particular, using endogenous miRNAs as backbones, artificial microRNAs (amiRNAs) exploit natural RNA silencing mechanisms to achieve the silencing of viral genes and in turn to generate resistance against different viruses [72].

The first amiRNA constructed using the miR159a precursor of *Arabidopsis thaliana (A. thaliala)* to confer viral resistance was reported by Niu et al. in 2006 [73]. In addition to the miR159a precursor in Arabidopsis, miRNA precursors including miR171a, miR172a, and mir528 have been modified to silence endogenous or exogenous targets and have been observed to be functional in Arabidopsis or tobacco [74,75,76,77]. The expression of different amiRNAs has demonstrated efficacy in different plants against a large variety of plant viruses [78,79]. Using *A. thaliana* miR167b and miR171a precursors as backbones rather than miR159a, an amiRNA-targeting sequence that encoded the silencing suppressor HC-Pro of PVY and p25 of PVX was designed and conferred high specific resistance against PVY and PVX infection in transgenic *Nicotiana tabacum (N. tabacum)*. This resistance was also maintained under conditions of increased viral pressure. The transgenic *N. tabacum* developed high effective resistance to both PVY and PVX through the expression of a dimeric amiRNA precursor. This indicates that amiRNA technology could be a promising tool with which to obtain multiple virus-resistance plants. Because of its exquisite specificity in avoiding off-target effects compared with long RNA-mediated silencing, amiRNA is considered a second-generation method and, with respect to viral immunity, also possesses the advantage of reducing potential biosafety-related risks when applied in agriculture.

To explore RNAi-directed viral resistance, expression cassettes carrying inverted repeats of PVS (genus *Carlavirus*) movement or CP sequences were used for generating viral-resistant plants against PVS, potato virus M (PVM), and PVY [61]. The results showed that transgenic lines representing seven cultivars remained free of any virus or only became infected with PVY. When progenies of transgenic lines of the cultivar Zeren were coinfected with PVS, PVM, and PVY, transgene-derived 21, 22, and 24 nt siRNAs were detected almost exclusively in the PVS inverted repeats. In some field progenies, 21–22 nt siRNAs from the entire PVY genome were detected. This indicates that transgenic RNAi is effective for virus degradation from naturally infected potato cultivars and protects from further infection in a sequence-specific manner [61].

Some secondary siRNAs are 21 nt phased siRNAs that are processed by successive DCL enzymes from the dsRNA substrate, which originates from an RDR from an AGO-catalyzed cleaved RNA at a miRNA target site [80,81]. Phased siRNAs are termed trans-acting siRNAs (tasiRNAs) [82] and are highly abundant in some plant families such as Solanaceae and Fabaceae but are not well conserved in other plant species [81]. TasiRNAs regulate plant development [83,84] and coordinate the repression of pentatricopeptide repeat (PPR) genes [85,86] or the nucleotide-binding site-leucine-rich repeat (NBS–LRR) family of resistance genes [87,88,89,90]. In the *A*. *thaliana* genome, families of genes coding for tasiRNA precursors (TAS) have been identified [82]. The TAS3 family is widely conserved in moss and higher plants and can generate tasiRNAs via a two-hit mechanism triggered by miR390 loaded in the specialized argonaute AGO7. The genes of the TAS1/TAS2 families, whose primary transcripts are targeted by a single hit of the 22 nt long version of miR173, are unique to Arabidopsis and are closely related species [91]. The miR173-triggered production of tasiRNAs has been used to engineer single or multiple copies of synthetic tasiRNAs (syn-tasiRNAs) to silence endogenous genes such as FAD2 [92], PDS [93], CH42 [94], and FT or TRY/CPC/ETC2 [95]. This syn-tasiRNA technology, named miRNA-induced gene silencing (MIGS), can reliably knock down single genes or multiple unrelated genes [96].

In natural infection, to protect themselves from plant RNA silencing systems, many viruses encode silencing suppressors to counteract host RNAi-based defenses. The first silencing suppressor, Hc-Pro, was discovered by three different groups independently in 1998. Since then, a large number of viral silencing suppressors have been identified, indicating that expressing proteins with RNA silencing activity is a common strategy used by plant viruses against RNA silencing in plants. Some silencing suppressors, such as HC-Pro, P38, P19, and P122, may interfere with RNA silencing amplification by binding small RNAs and by preventing secondary siRNA accumulation, while other silencing suppressors directly interact with AGO protein and suppress the silencing system. AGO proteins appear to be targeted by silencing suppressors in different ways.

The second layer of the antiviral component in RNA silencing is AGO proteins. Some AGO proteins, such as AGO1, AGO2, and AGO7 in Arabidopsis and AGO2 and AGO4 in *N. benthamiana*, are involved in the antiviral effect. The counter-defense role of P25 is directed by the degradation of AGO proteins through the proteasome pathway [97]. It was demonstrated that the amount of AGO1 in infiltrated leaves carrying P25 was dramatically decreased compared with those infiltrated with HC-Pro, but it could be restored when treated with the proteasome inhibitor MG132. Plants treated with MG132 were less susceptible to PVX and its relative bamboo mosaic virus [97].

In most cases, viral silencing suppressors are strong enough to counteract RNAi and result in viral infection in plants. To confer high viral resistance, researchers should therefore focus on how to improve RNAi activity by increasing the efficiency of AGO proteins first by modifying siRNA, that is, by facilitating loading into the RISC complex. Modifying siRNA near the 5′ termini could improve RNAi activity and the strand selectivity of RISC formation. Virus-derived siRNAs are active in targeting viral mRNA. Thus, it is advantageous to improve the ability of RISC to recruit vsiRNAs and to exert the cleavage of target viral mRNA. Second, AGOs should be modified in changing the status of AGOs from inactive to active and from slicer to translation inhibition. Great efforts have been made to define AGO functions by the selection of specific defective mutant alleles based on protein structure. This is very helpful in understanding how the AGO family plays a role in regulatory functions in plant biology. Researchers should also focus on modifying inactive AGO proteins and changing them into active AGO proteins or changing their function from slicer to translation inhibition.

In the mammalian system, it has been observed that AGO proteins can be post-translationally modified such as with modifications in hydroxylation, phosphorylation, and ubiquitylation, influencing Argonaute stability and function [98,99,100]. However, AGO modifications are not yet clear in plants. Future research should work toward unraveling novel AGO modifications in plants and their corresponding functions. A strategy based on increasing expression levels of AGOs to meet requirements of AGO-mediated resistance could also be considered. This may also prove significant because low-expressed AGO proteins engineered to express at high levels would be useful in facilitating research and in helping us to find new functions of AGOs.

In addition, another open question is how AGO proteins collaborate with other plant defense pathways to confer an antiviral effect. The crosstalk between RNA silencing and plant immune systems remains unexplored. It has been proposed that RDR1 might play a dual role, firstly contributing to salicylic acid-mediated antiviral defense and secondly suppressing RDR6-mediated antiviral RNA silencing [81]. This suggests that RNA silencing may collaborate with other plant defense systems, which is supported by virus resistance induced by NB–LRR proteins involving AGO4-dependent translational control.

Even though the role of RNA silencing in antiviral plant defense has been well studied, the positive effect of RNA silencing in viral infection remains unknown. It is possible that some components of RNA silencing systems could directly or indirectly contribute to viral infection. It was discovered that DCL4 may have a positive effect on PSTVd accumulation in *N*. *benthamiana*, while DCL2 does not [101]. The mechanism of this protecting effect is still not clear.

In summary, to establish successful infection, plant viruses suppress or evade RNAi and other innate immunity systems that crosstalk with RNAi [102,103], which offers us several possibilities for engineering viral resistance in potato.

## 5. CRISPR/Cas9-Mediated Viral Resistance in Potato

CRISPR/Cas (clustered regularly interspaced short palindromic repeats/CRISPR-associated) is derived from the genomes of bacteria, and its original function was to provide bacteria with specific immune protection against invading nucleic acids [104]. This system became a powerful tool for genome engineering, which enables the efficient modification of endogenous genes in various species and viral disease resistance traits [105,106,107]. There are now increasing reports demonstrating that CRISPR/Cas systems can be harnessed to develop antiviral immunity in plants with high efficiency [108,109,110]. sgRNA-Cas9 constructs targeting beet severe curly top virus (BSCTV), which inhibits virus accumulation in *N. benthamiana* and *A. thaliana* [84]. Moreover, viral resistance could be obtained through the CRISPR/Cas9 editing of plant endogenous genes. Mutated eIF4G alleles in rice were generated using the CRISPR/Cas9 system in the RTSV-susceptible variety IR64, widely grown across tropical Asia, and conferred resistance. The Cas9 sequence did not exist in the final products with RTSV resistance, and the yield was enhanced under glasshouse conditions [111].

Several studies have introduced the generation of virus-resistant potato crops using CRISPR-mediated technology. Zhan and colleagues generated potato-virus-Y-resistant potatoes with CRISPR/LshCas13a [112]. A correlation between the level of resistance and the degree of Cas13a/sgRNA expression was observed. It was reported that the Va gene (Ntab0942120) in tobacco determines the susceptibility of the plant to PVY [113]. The Va gene product interacts with the PVY genome-linked protein (VPg) to initiate the PVY genome translation process, which ultimately leads to the systemic infection of tobacco by the virus [114]. The Va gene in tobacco cultivar LJ911 was knocked out via CRISPR/Cas9 technology. Edited plants showed PVY resistance [113]. These reports demonstrate the potential of CRISPR/Cas9 in editing susceptibility genes to obtain antiviral immunity for controlling plant RNA viruses in potato.

## 6. Future Prospects and Conclusions

Although great progress has been made in molecular virus–host interactions, due to most potato cultivars lacking broad-spectrum resistance to genetically complex strains of viruses, further efforts are required to explore viral resistance. In the future, several strategies might assist in obtaining broad-spectrum resistance:

(i) Disrupting the interaction between the virus and host through potato genome editing will efficiently protect potato from viral infection. The available potato genome sequences (Potato Genome Sequencing Consortium 2011) will facilitate such studies. Instead of RNAi, CRISPR-editing-mediated antiviral immunity might be a versatile technology with which to combat plant virus infections [107].

(ii) Discovering resistance genes that are important to antiviral defense will offer great opportunities for potato breeding. Identified resistance genes may also be introduced to potato via genetic transformation.

(iii) Manipulating inducible defense in plants that are naturally resistant to viruses might be an effective approach for potato breeding. Plant defenses have broad-spectrum capabilities. Recently, much evidence has supported the identification of viral components that trigger plant immune mechanisms. This will become a popular research area wherein the resistance genes that control these defense mechanisms may be identified. It will be possible to design methods of engineering the broad-spectrum components of natural defense mechanisms.

(iv) Based on the increasing understanding of the molecular functions of viral proteins, especially those related to replication and virus movement, in the future, we may manipulate viral proteins used for inoculums to obtain cross-protection from further viral infection in potato.

(v) The transgenic expression of antiviral proteins of non-plant origin, including antibodies, may also represent a promising approach with which to obtain resistance to specific potato viruses.

## Figures and Tables

**Table 1:** Viral resistance gene and location in potato chromosome.

Name of Resistance Gene	Virus	Source	Chromosome	Reference
Ry_sto_	PVY	I-1039 *S. stoloniferum*	XI	Brigneti (1997) [53] Song (2005) [29]; Flis (2005) [28]
Ry_adg_	PVY	*S. andigena,* line 2X(v-2)7	XI	Hämäläinen (1998) [23]
Ry_chc_	PVY	Japanese leading cultivar ‘Konafubuki’	IX	Masatoshi Sato (2006) [31]
Ny_tbr_	PVY	USW2230	IV	Celebi-Toprak (2002) [45]; Benoît Moury (2011) [46]
Nc_tbr_	PVY	*S. tuberosum*	IV	Benoît Moury (2011) [46]
Nc_spl_	PVY	*T. tuberosum*	IV	Benoît Moury (2011) [46]
Ny-1	PVY	Rywal and Accent	IX	Szajko (2008) [24]; Szajko (2014) [54]
Ny-2	PVY	Romula	IX	Szajko K (2014) [54]
Y-1	PVY	*S. tuberosum* ssp. *andigena*	XI	Vidal (2002) [38]
*G-Ry*	PVY			Lee (2010) [40]; Vidal (2002) [38]
Nx_phu_	PVX	phu Iv35	IX	Tommiska (1998) [55]
Rx(Rx_adg_)	PVX	*tbr cv.Cara*	XII	Bendahmane (1997) [56]
Rx1	PVX	*S. andigena*	XII	Ritter (1991) [57]
Rx2(Rx_acl_)	PVX	*S. acaule*	V	Ritter (1991) [57]
PLRV.1	PLRV	DG83-68	XI	Marczewski (2001) [52]
PLRV.2	PLRV	DG83-2025	VI	Marczewski (2001) [52]

## Data Availability

Not applicable.
